# Digital PCR linkage analysis resolves *Streptococcus pneumoniae* signature from commensal interference in saliva samples: identifying wolves among sheep in wolf’s clothing

**DOI:** 10.1128/spectrum.03131-25

**Published:** 2026-03-25

**Authors:** Willem R. Miellet, Tessa Nieuwenhuijsen, Eline H. M. R. van den Oetelaar, Janieke van Veldhuizen, Nynke Y. Rots, Krzysztof Trzciński, Rob Mariman

**Affiliations:** 1 National Institute of Public Health and the Environment (RIVM), Bilthoven, the Netherlands; 2 University Medical Center Utrecht, Utrecht, the Netherlands; Friedrich-Alexander-Universitat Erlangen-Nurnberg, Erlangen, Germany

**Keywords:** digital PCR, linkage, *Streptococcus pneumoniae*, saliva, serotyping, vaccine

## Abstract

Serotyping of *Streptococcus pneumoniae* (pneumococcus) is essential for evaluating the effects of conjugate polysaccharide vaccines on asymptomatic colonization (carriage) of vaccine-targeted capsular variants (serotypes). However, culture-independent application of molecular methods is hampered by genetic exchange between pneumococcus and oral streptococci co-residing within the airways. Unlike quantitative PCR (qPCR), digital PCR (dPCR) enables linkage analysis that allows for the identification of gene co-occurrence within individual bacterial cells. To minimize false-positive classification with molecular serotyping of highly polymicrobial saliva samples, a duplexed dPCR protocol for linkage analysis was developed. The performance of the protocol was evaluated by determining linkage between *piaB* and *lytA,* as well as between *piaB* and genes coding for serogroups 6, 9, and serotype 4 capsular polysaccharides in 141 saliva samples from children and adults from children and adults residing in the Netherlands, to identify pneumococcal strains present during natural carriage. Identification of linkage between distant genes required intact pneumococcal cells. The 95% limit of detection for linkage analysis between *piaB and lytA* was determined to be 9.2 CFU/reaction. Co-occurrence of *piaB* and *lytA* within single bacterial cells was consistently observed in saliva samples. Similarly, linkage was identified between serogroup 6 and *piaB* in the majority of saliva samples from serogroup 6 carriers. Application of dPCR distinguished pneumococcus from oral *Streptococcus* species in saliva tested for serogroup 9 and serotype 4. Duplex dPCR linkage analysis allowed for differentiation of pneumococcus from oral streptococci with homologous capsular loci. By improving the diagnostic accuracy of molecular surveillance, linkage analysis is a promising technique for culture-independent epidemiologic surveillance of pneumococcal serotype carriage.

**IMPORTANCE:**

Accurate monitoring of pneumococcal conjugate vaccine effects requires reliable detection of *Streptococcus pneumoniae* serotype carriage in community-dwelling populations. However, sequences shared between pneumococcal strains and related commensal oral streptococci complicate this procedure. Here, we describe a method that takes advantage of the unique capacity of digital PCR (dPCR) to identify non-random associations among molecular targets in a sample, indicative of a single cell origin. Proof of principle was demonstrated using mock bacterial communities, followed by application to 141 saliva specimens from community-dwelling individuals. This methodological advance provides a reliable means of determining serotype-specific *S. pneumoniae* carriage using oral fluids, an approach particularly valuable for vaccine impact studies in adult populations. Given the growing public health interest in adult pneumococcal vaccination programs and the lack of validated methods for oral mucosa, we believe our protocol represents a timely and important contribution to the field. In addition, our findings help resolve longstanding debates on the utility of oral fluids for pneumococcal surveillance.

## INTRODUCTION

*Streptococcus pneumoniae* (pneumococcus) is a commensal bacterium of the human upper respiratory tract (URT) but also a major cause of disease, with the highest incidence in young children and older adults. The polysaccharide capsule is considered to be the primary virulence factor of pneumococcus. Over 100 distinct capsular polysaccharide serotypes have been identified (1). Capsular polysaccharides are also the antigens in all currently marketed pneumococcal vaccines (1). Most broadly used vaccines are pneumococcal conjugate vaccines (PCVs) that prevent not only disease but also acquisition of carriage with vaccine-targeted (VT) serotypes (2). Consequently, the carriage of these serotypes is accepted as an endpoint in vaccine impact studies (3).

Among young children, colonization rates of both the nasopharyngeal and oral mucosa are high (4–7). Due to high absolute and relative abundances of *S. pneumoniae* within the nasopharynx, it is relatively straightforward to detect pneumococcal carriage through culture of nasopharyngeal samples in this age group. In contrast, culture-based methods have proven inadequate for the sensitive detection of *S. pneumoniae* at highly polymicrobial sites, such as the oropharyngeal and oral mucosa (8–12). This is particularly problematic in adults, among whom pneumococcal colonization of the upper respiratory tract is largely restricted to the oropharynx and oral cavity (13). Disparity between culture and culture-independent methods for such samples mirrors the “great plate count anomaly” due to the inadequacy of culture-based methods when applied to highly polymicrobial samples (14). Consequently, oral and oropharyngeal samples are often overlooked for pneumococcal carriage detection.

Molecular diagnostic methods can be used to accurately identify *S. pneumoniae* and pneumococcal serotypes carriage in individuals across various age groups (15). However, the utility of molecular diagnostic assays to oral samples is at times complicated by habitat overlap of *S. pneumoniae* and other oral streptococci (*S. mitis* and *S. oralis*, etc.) and frequent genetic exchange between these bacteria (16, 17). Although recent advances in quantitative PCR (qPCR)-based carriage surveillance have enabled sensitive evaluation of diagnostic accuracy through the application of the Two-to-Tango approach (12, 15), interspecific horizontal gene transfer has rendered numerous qPCR assays unreliable in multiple geographic settings, including assays designed to detect serotypes that are targeted by licensed PCVs (15). Like sheep in wolves’ clothing, the circulation of such non-pneumococcal streptococci with homologous capsular operons has complicated the interpretation of molecular serotyping results. Consequently, while we and others have defined certain limitations of culture-independent surveillance methods (15, 18–22), some of our colleagues have altogether discouraged the application of carriage surveillance on oral samples (1, 23), and culture-independent testing of oral fluids is currently not recommended by the World Health Organization Pneumococcal Carriage Working Group (24).

Here, we describe a digital PCR (dPCR) based protocol capable of confirming *S. pneumoniae* as the source of serotype signature in polymicrobial samples. By partitioning duplexed molecular diagnostic tests into many micro reactions, it becomes possible to identify whether two targets are non-randomly linked, either physically on the bacterial genome or as part of the same bacterial cell (e.g., bacterial chromosome and plasmid). While unlinked targets distribute randomly across partitions, linked targets tend to co-localize consistently in the same partitions, resulting in a high ratio of double-positive to single-positive partitions. By using intact bacterial cells as input for dPCR (25, 26), we assessed linkage between physically distant targets to address specificity limitations of various serotype/serogroup-specific PCR assays, namely serotype 4, serogroup 6 (6A/B/C/D), and serogroup 9 (9A/L/N/V). We validated this approach by using suspensions of mixed cells of defined streptococcal strains. Finally, we demonstrated the utility of the protocol by applying it to 141 culture-enriched saliva samples collected from community-dwelling individuals to detect naturally occurring pneumococcal carriage strains.

## MATERIALS AND METHODS

### Microbial strains

All bacterial strains used in this study are listed in Table S1, with lab materials also listed in Table S2. A *Streptococcus sanguinis* cultured from the upper airways in the carriage study and found by qPCR to be positive for serogroup 9 capsular operon (*cps*) isolate, yet negative for *piaB* and *lytA* (strain Sm226001019702), was used alongside *S. pneumoniae* strain P2007-1850 (serotype 9A) to evaluate the performance of duplex dPCR in detecting gene linkage in mixed streptococcal samples. For comparison of DNA extract versus intact cell input and as a positive control in *piaB-lytA* duplex assays, the *S. pneumoniae* strain Hungary 19A-6 (serotype 19A) was used. Strains PI2018-0103 (6A), PI2013-2681 (6C), P2007-1850 (9A), and PI2018-0984 (4) were included as serotype-specific positive controls in dPCR analyses targeting each respective serotype together with *piaB*.

### Human subjects

Pneumococcal carriage was investigated in a cross-sectional prospective observational study conducted in 2015/2016 in the Netherlands (27).

### Sample collection and laboratory processing

As described previously (10), saliva samples were collected from all individuals with a sponge lollipop (Oracol Saliva Collection System, Malvern Medical Developments Limited, Worcester, UK), immediately transferred to tubes pre-filled with 100 μL sterile 50% glycerol solution in water, mixed, placed on dry ice, and transported to the lab. With approximately 400 μL of saliva collected per sample, the final glycerol concentration was around 10%. Saliva samples were delivered to the laboratory and stored at −70°C within 8 h. For dPCR analysis, saliva samples from children and adults were included if they were previously identified as positive for both *piaB* and *lytA*, and, in cases involving molecular serotyping, also positive for the relevant serotype qPCR assay (5). Where applicable, these results were compared with Quellung-based serotyping performed on pneumococcal strains isolated from paired nasopharyngeal swabs (12). In addition, selected samples were chosen to span a C_q_ range representative of the full sample set.

### Study sample culture enrichment

Glycerol-supplemented saliva was used to inoculate gentamicin sheep blood agar (SB7-Gent agar, Oxoid). After overnight incubation at 37°C and 5% CO_2_, all growth was harvested from a plate into 2.1 mL of brain heart infusion broth (BHI; Oxoid) supplemented with 0.5% yeast extract (Oxoid) and 10% glycerol and stored at −70°C (5). To maximize the abundance of viable pneumococcal cells in cultures, harvests from SB7-Gent plates were used to inoculate Columbia blood agar (CBA) with 5% defibrinated sheep blood and incubated for 6 h at 37°C with 5% CO_2_, and all colony growth was harvested from a plate and stored as described above.

### Bacterial cell suspensions and CFU assessment

*S. pneumoniae* positive controls were prepared by growing the strains in BHI with 0.5% yeast extract to mid-logarithmic growth phase (OD_620_ of 0.1) at 37°C with 5% CO_2_. The cultures were then placed on ice, and glycerol was added to a 10% final concentration before aliquoting and storing cell suspensions at −70°C. Five eightfold serial dilutions were prepared from an aliquot in phosphate-buffered saline, and the fourth and fifth dilutions were plated on CBA in duplicate. After overnight incubation at 37°C with 5% CO_2_, colony counts were used to calculate the concentration in CFU/mL, which was used to assess the dynamic range of the dPCR linkage protocol and estimate the 95% limit of detection (LoD_95_).

### Primers and probes

Primers and probe targeting sequences within genes coding for the pneumococcal iron uptake ABC transporter lipoprotein PiaB (8), and for the major pneumococcal autolysin LytA (28) were used to quantify pneumococcal DNA in cell suspensions and nucleic acid extracts. We used three assays for molecular serotyping in combination with *piaB*, namely assays targeting the serogroup 6ABCD (*wciP* gene), 9ALNV (*mnaA* gene), and serotype 4 (*wzy* gene) *cps*. Primers and probes used in these assays and their concentrations are listed in Table S3.

### Nucleic acid extraction and quantitative PCR

As described previously (5), DNA was extracted from culture-enriched saliva by centrifuging 100 μL of plate harvest (2 min, 14,000 × *g*), resuspending the pellet in 90 μL TE buffer (20 mM Tris-HCl, 2 mM EDTA, pH 8.0), and incubating at 95°C for 15 min. After adding 90 μL lysis buffer (20 mM Tris-HCl, 2 mM EDTA, 2.4% Triton X-100, 40 mg/mL lysozyme), DNA was purified using the DNeasy Blood & Tissue Kit (Qiagen) and eluted in 200 μL buffer. Next, 1.0 µL DNA extract within a total reaction volume of 12.5 µL with SensiFast Probe No-Rox mastermix (BIO-86050, Bioline) was tested with a LightCycler480 qPCR unit (Roche). The thermocycler conditions were as follows: one cycle of 95°C for 10 min, followed by 45 cycles of 95°C for 10 s and 60°C for 45 s. C_q_ calling was performed according to manufacturer instructions with a standard curve and using the second derivative maximum method.

### Digital PCR

dPCR was performed using the QIAcuity One 2plex dPCR instrument (cat.nr. 911000, Qiagen) according to the manufacturer’s recommendations. Reactions of 40 µL were prepared with QIAcuity Probe mastermix (cat.nr. 250101, Qiagen) and loaded in a QIAcuity 26k 24-well plate (cat.nr. 250001, Qiagen) in partition volumes of approximately 0.82 nL. Unless stated otherwise, the thermocycler conditions were as follows: QIAGEN Priming Profile Probe (RT-) PCR, one cycle of 95°C for 5 min, followed by 55 cycles of 95°C for 30 s and 60°C for 60 s. The imaging settings of duplex reactions used for linkage analysis are listed in Table S3. All plates included no-template controls and a positive control (either DNA extract or log-phase cell suspension of a pneumococcal strain). Study samples were diluted prior to testing based on prior qPCR results: samples with *piaB*/serotype C_q_ values <22 were diluted 1:1,000, those with C_q_ values between 22 and 25 were diluted 1:100, and those with C_q_ ≥25 were diluted 1:10. All samples were tested in singlicate.

### Baseline correction and RFU thresholding

Relative fluorescence unit (RFU) thresholds were derived using the method described by Trypsteen et al. based on extreme value theory (29). Prior to RFU thresholding, baseline correction was conducted as outlined in the same study (29). In short, all no template controls measurements from a particular duplexed assay were combined and split into blocks of 150 partitions. The initial thresholds were set to the 0.9995 quantile, and the procedure was iterated 10,000 times. Estimated RFU thresholds were thereafter manually curated, and in case of inaccurate RFU thresholding, initial thresholds and block size were adjusted.

Details related to the Minimal Information for MIQE Experiments (dMIQE) guidelines for digital droplet PCR reporting are outlined in Table S5 (30).

### Linkage analysis

Concentration-dependent linkage (linkage for short) was calculated according to the method described by Regan et al. (31). As these values are inherently influenced by target concentration and given the typically low abundance of *S. pneumoniae* in oral fluids, concentration-independent linkage was also calculated for each target within a duplex reaction using the following approach. If two targets are unlinked, their distribution among partitions follows the Poisson distribution:


$$N_{A}\times \;N_{B}=N_{E}\times \;N_{AB}.$$

Where *N* indicates the number of partitions analyzed, with *N_A_* and *N_B_* the number of partitions positive for a single target, *N_AB_* the number of double-positive partitions, and *N_E_* the number of double-negative partitions. By rearranging the equation, we can then calculate the number of double-positive partitions by random chance (*N_Ch_*):


$$N_{Ch}=\frac{N_{A}\times \;N_{B}}{N_{E}}.$$

When linkage is observed within a reaction, *N*_AB_ *> N*_Ch_. For such samples, the number of partitions attributable to linkage (*N_L_*) can be calculated with


$$N_{L}=N_{AB}-N_{Ch}.$$

Using this information, we can calculate concentration-independent linkage values via the following formulas:


$$\;\;\;\;\;\;\;\;\%\;linkage\;A=\left(N_{AB}-N_{Ch}\right)/\left(N_{AB}+N_{A}\right)\times 100,$$

$$\;\;\;\;\;\;\;\;\%\;linkage\;B=\left(N_{AB}-N_{Ch}\right)/\left(N_{AB}+N_{B}\right)\times 100.$$

Concentration-independent linkage values were subsequently used for all analyses. Unless stated otherwise, linkage values are reported with *piaB* as the reference target. A sample was classified as positive for linkage when *N_L_* was ≥5, and a two-tailed Poisson test (with α = 0.01) indicated linkage, meaning that the 99% confidence interval of *N_AB_* did not include *N_Ch_*.

### Bland-Altman analysis

Presence or absence of linkage in relation to mean target concentration and target agreement was illustrated using Bland-Altman plots. Non-parametric limits of agreements were used to visualize the degree of agreement in dPCR measurements between duplexed targets, to assess potential bias, defined as the ratio of copies per microliter (cp/µL) of assay A to that of assay B (32).

### Probit regression analysis

The LoD_95_ was derived by fitting a probit regression model to the counts of successful and failed detections of linkage at specific CFU/mL concentrations. This was done using the *glm* function from the “stats” R package, with a binomial family and probit link.

All statistical analyses were performed in R v4.4.3 using RStudio (2025.05.0-496).

## RESULTS

### Pneumococcal cell suspensions, but not DNA extracts, display linkage between two targets

**Fig 1:**
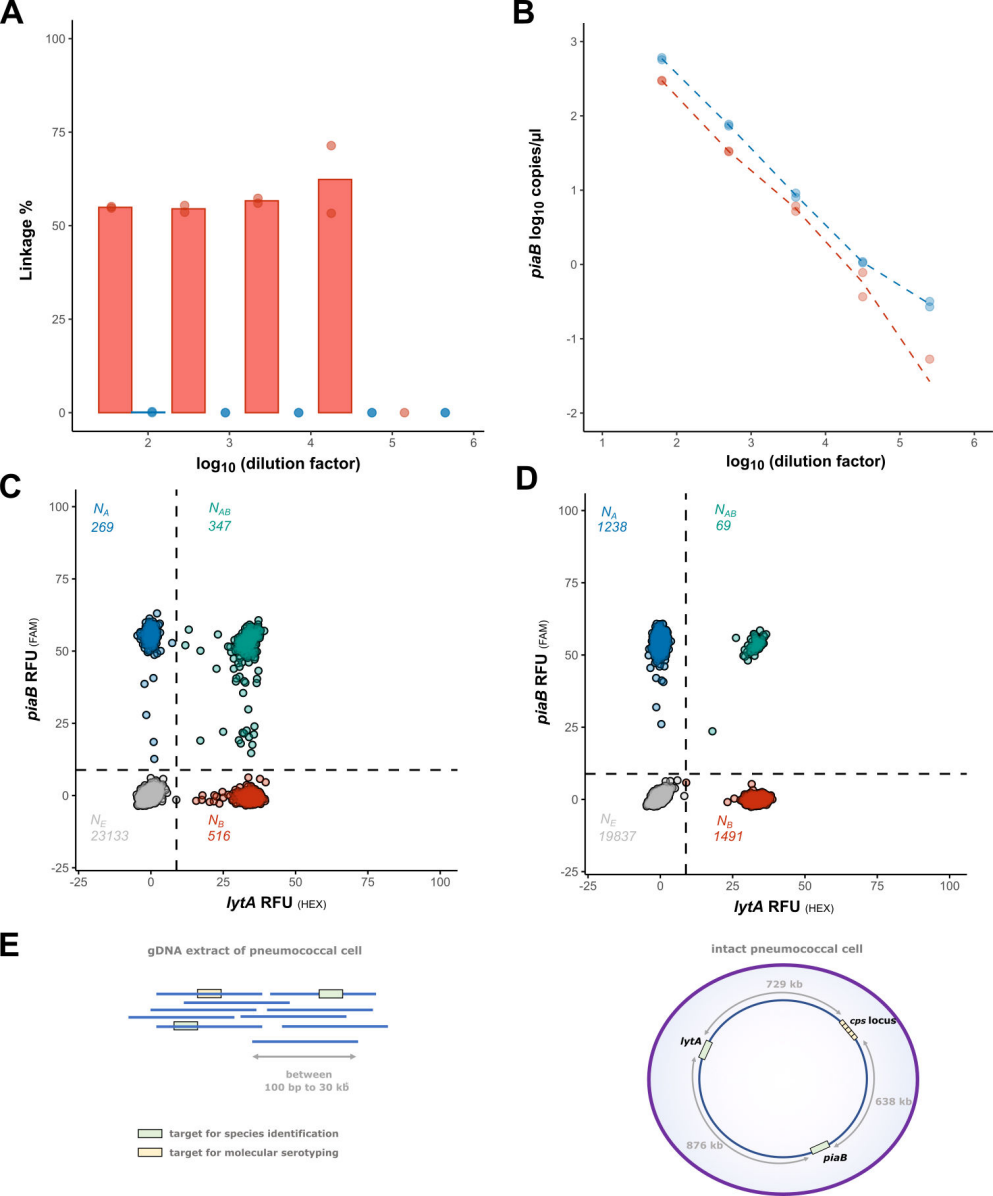
Linkage between *piaB* and *lytA* for intact cells and DNA extracts of *S. pneumoniae* Hungary 19A-6 strain. (**A**) Linkage observed with intact cells (red) and DNA extracts (blue) at a given concentration. Utilizing intact bacterial cells as input material permits the identification of linkage between targets that are genomically distant, while linkage between distant targets is lost when DNA extracts are used. Linkage % estimates were calculated with *piaB*-positive partitions as the denominator. (**B**) Quantification of *piaB* with intact cells (red) and DNA extracts (blue) at a given dilution of an input sample. Bacterial cell suspensions were 1.87E^2^ to 6.12E^6^ CFU/mL, and DNA extracts were 3.5E−2 to 1.1E−6 ng/µL, equivalent to 0.3 to 793 cp/µL. (**C**) Representative 2D plot of bacterial cell suspension of 14 cp/µL (*piaB*), the ratio of double-positive to single-positive partitions is suggestive of linkage (347 × 23,133 > 269 × 516). (**D**) Representative 2D plot of DNA extract of 32 cp/µL (*piaB*). The ratio of double-positive to single-positive partitions was not indicative of linkage (69 × 19,837 ≈ 1,238 × 1,491). (**E**) Linkage detection between genetic loci relies on either the genomic distance or the physical proximity of targets. If targets are separated by more than the average DNA fragment length, co-amplification becomes stochastic.

Using dPCR, we subjected suspensions of intact cells and DNA extracts of the *S. pneumoniae* Hungary 19A-6 strain to duplex quantification to assess the linkage between two genes, *piaB* and *lytA*, a combination of targets presumed to be highly specific for pneumococcus (33). In both types of samples, quantification of both molecular targets was observed (Fig. 1B). Suspensions with intact pneumococcal cells consistently produced partitioned reactions with co-amplification of *piaB* and *lytA* beyond a number expected due to chance (Fig. 1A and C). Such results were interpreted as evidence of linkage between two genes, meaning both genes were derived from a single bacterial cell. By contrast, duplex dPCR reactions with DNA extracts exhibited no linkage between the two targets (Fig. 1A and D). The number of partitions with co-amplification of *piaB* and *lytA* in these reactions corresponded with the number expected due to chance (Fig. 1D). Absence of linkage with DNA extracts was hypothesized to be due to the considerable distance between *piaB* and *lytA* in the typical pneumococcal genome. In the *S. pneumoniae* Hungary 19A-6 strain, these two genes are approximately 876 kb apart, a distance exceeding the maximum fragment size of genomic DNA extracts that are typically ~30 kb (Fig. 1E).

### Critical determinants for linkage analysis

It was reported that raising the number of PCR cycles, pre-treating bacterial cells with lysozyme, or supplementing reaction mixtures with bovine serum albumin (BSA) concentration may lead to an increase in percentage linkage or improved PCR performance (34–36). The impact of PCR cycle number on linkage was tested by a stepwise increase by 5 cycles from 40 recommended by the manufacturer to 60 (data not shown). Raising the number of cycles to 55 was associated with a modest increase in percentage linkage. Neither treatment of pneumococcal cells with lysozyme nor increasing the concentration of BSA resulted in an increase in linkage (data not shown). Of note, cell suspensions were tested exclusively on 26k well plates, as the 8.5k well plates were prone to clogging, thus rendering them unsuitable for dPCR with whole bacterial cells (data not shown).

### Analytical sensitivity estimation via probit regression analysis

The analytical sensitivity of the protocol for detecting linkage was evaluated using probit regression with a twofold serial dilution of *S. pneumoniae* Hungary 19A-6 strain cells, ranging between 1.20E+04 and 1.87E+02 CFU/mL, with 12 replicates tested for each dilution. LoD_95_ was estimated to be 1,664 CFU/mL, which corresponded to 9.2 CFUs per reaction or 0.8 copies/µL (Fig. S1). Importantly, to minimize false-positive linkage detection in samples with faint PCR target quantification, a sample was deemed positive for linkage when a minimum of five partitions showed co-amplification not attributable to chance (*N*_*L*_), and a Poisson test of observed (*N*_AB_) versus expected partitions with co-amplification (*N*_Ch_) indicated linkage (*N*_AB_ > *N*_Ch_).

### In mixtures of streptococcal strains, dPCR-based linkage is observed only if *S. pneumoniae* is present

**Fig 2:**
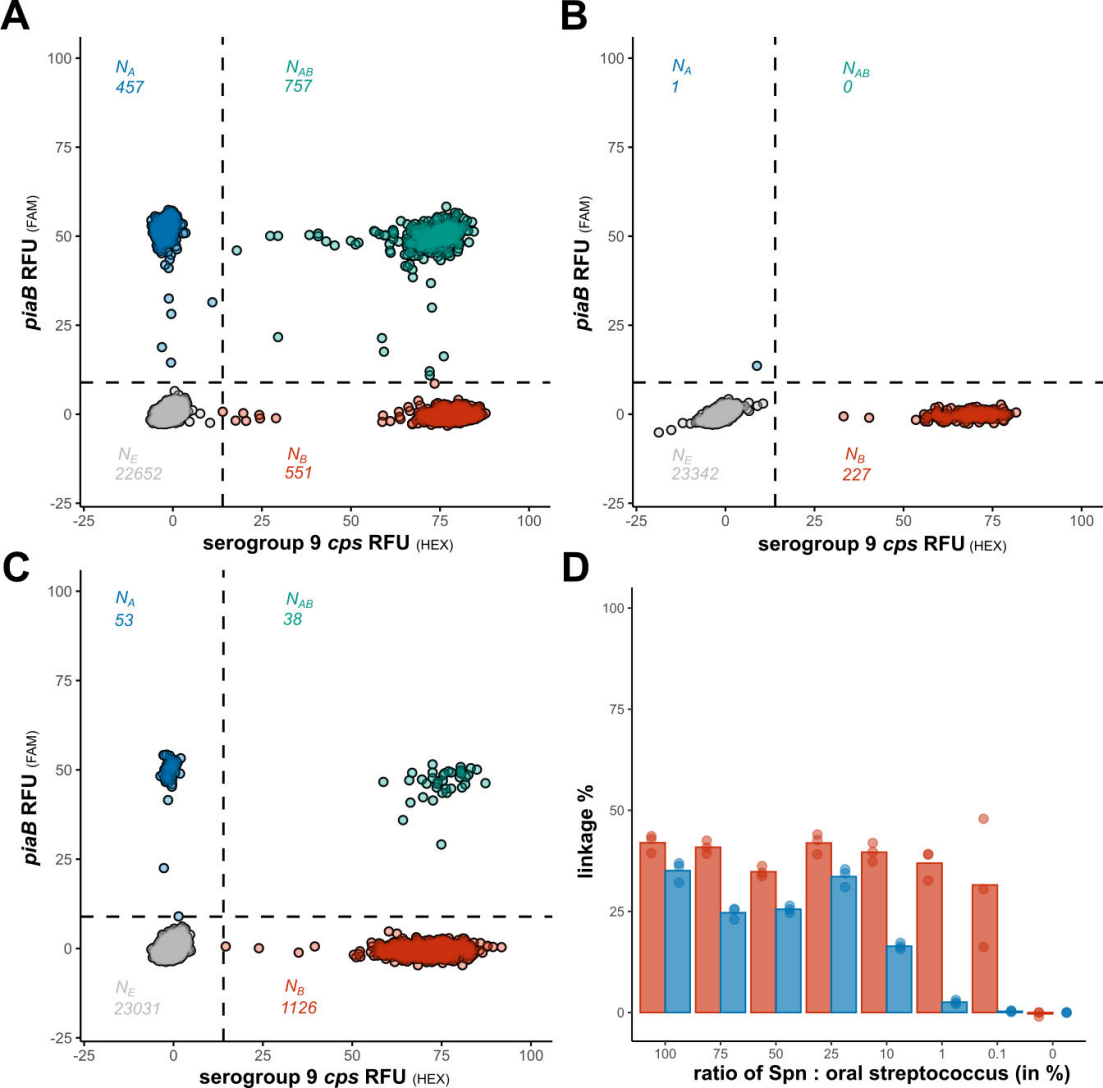
dPCR resolves the *S. pneumoniae* serogroup 9 *cps* signature in mixed suspensions containing both *S. pneumoniae* and *S. sanguinis* strains. (**A**) A 2D plot of the serogroup 9 *cps S. pneumoniae* strain with linkage evident between *piaB* and the serogroup 9 *cps* assay. (**B**) A 2D plot of the serogroup 9 *cps S. sanguinis* strain, which only produced quantification for the serogroup 9 *cps* assay and not for *piaB*, hence no linkage was evident. (**C**) A 2D plot of a suspension with a 1:100 ratio of serogroup 9 *cps S. pneumoniae* strain and *S. sanguinis* serogroup 9 *cps* strain, linkage between *piaB* and serogroup 9 *cps* assay remains identifiable. (**D**) Bar diagram of percentage linkage of suspensions of serogroup 9 *cps S. pneumoniae* strain mixed with *S. sanguinis* serogroup 9 strain in various ratios. Red and blue bars indicate linkage percentage based on *piaB* and serogroup 9 *cps* assay, respectively.

To emulate the complexity of polymicrobial samples with mixtures of pneumococci and oral streptococci, we evaluated the performance of the protocol applied to bacterial cell suspensions with varying ratios of serotype 9A *S. pneumoniae* strain P2007-1850 (Fig. 2A) to *S. sanguinis* Sm2260010119702 (Fig. 2B). Both strains were positive by qPCR for the *mnaA* gene unique to serogroup 9, therein further described as serogroup 9 *cps* assay*,* yet only the *S. pneumoniae* was positive for *piaB* (Table S2). Linkage between *piaB* and serogroup 9 *cps* was only detectable in mixes containing *S. pneumoniae* cells, provided the pneumococcal abundance was above the LoD_95_ (Fig. 2D). Linkage remained detectable in mixes where the *S. sanguinis* strain exceeded pneumococcus by up to 100-fold (Fig. 2C). In these mixes, the percentage of linkage estimated by *piaB* remained consistent, while estimated by the serogroup 9 *cps* target decreased with increasing relative abundance of the *S. sanguinis* strain (Fig. 2C and D). Taken together, linkage remained identifiable in mixes, supporting the suitability of the protocol for testing complex polymicrobial samples such as saliva.

### Assessment of linkage in saliva study samples and optimization of culture conditions

**Fig 3:**
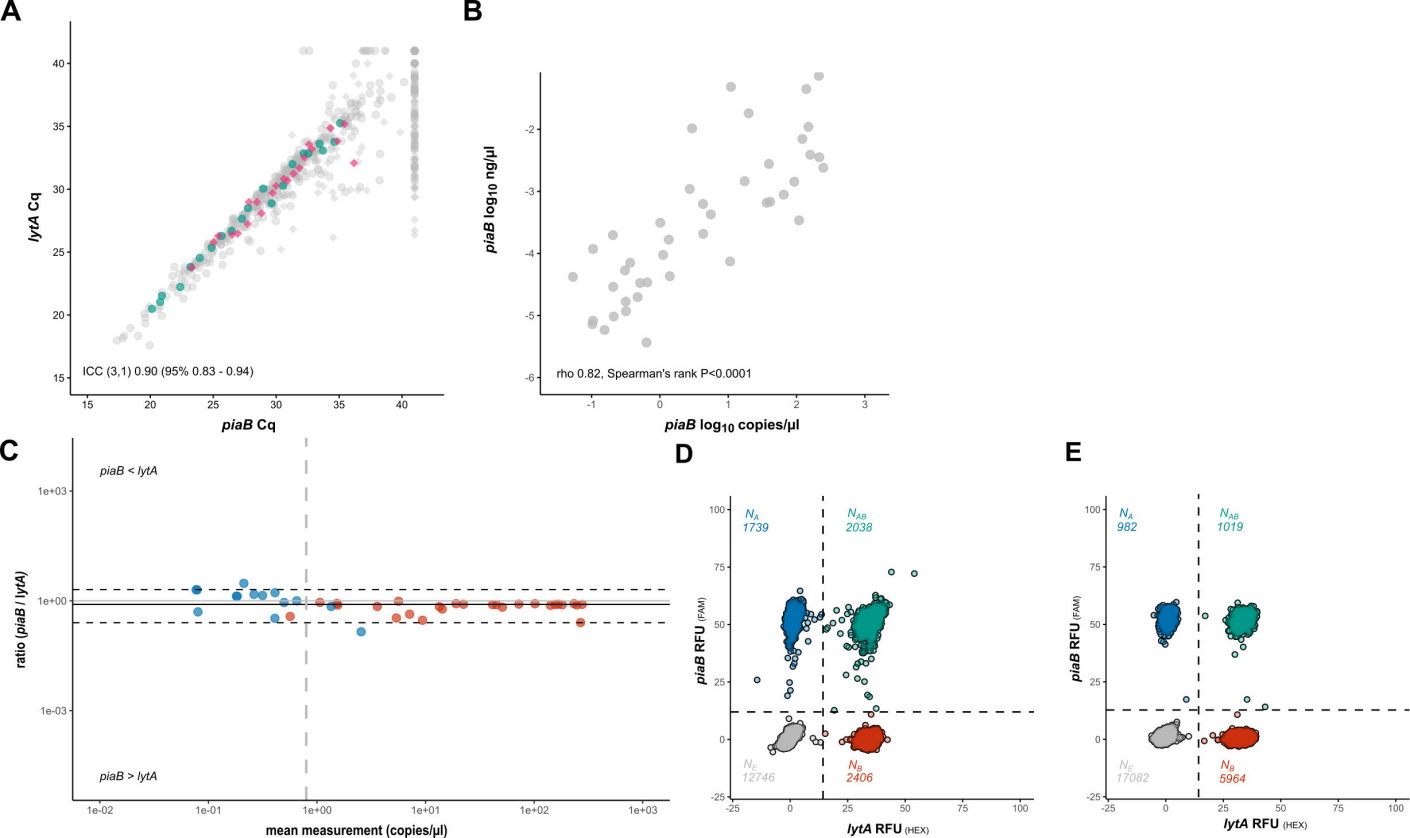
dPCR-based linkage analysis provides evidence for positive conditional dependence of *piaB* with *lytA* signal in culture-enriched saliva samples, which is associated with *S. pneumoniae* presence. (**A**) qPCR-based quantification (in C_q_) of *piaB* and *lytA* in DNA extracts of culture-enriched saliva study samples. Samples from children and adults that were selected for dPCR-based linkage analysis are highlighted in green and pink, respectively. The remaining samples are colored gray. Circles and diamonds indicate samples from children and adults, respectively. (**B**) The correlation between cultures suggests that pneumococcal presence, as measured by *piaB*, was retained in saliva cultures after preparation of 6-h cultures (dPCR) from the original overnight cultures (qPCR). (**C**) Non-parametric Bland-Altman plot of dPCR-based quantification (in copies/µL) *piaB* and *lytA* in culture-enriched cell suspensions, samples positive for linkage between *piaB* and *lytA* are highlighted in red, and negative samples are highlighted in blue. The vertical line indicates the LoD_95_, dashed horizontal lines the limits of agreement, and the solid line the bias (median difference between paired *piaB* and *lytA* measurements). (**D**) An example of a 2D dPCR plot wherein linkage to a relatively high number of partitions with co-amplification of *piaB* and *lytA* is observed, a result suggestive of linkage. (**E**) An example of a 2D dPCR plot showcasing linkage between *piaB* and *lytA* and concurrent presence of oral *Streptococcus* (or non-typeable pneumococcus), which is exclusively positive for *lytA*.

In the experiments that followed, saliva samples collected from community-dwelling individuals were tested with dPCR for linkage between pneumococcal genes present during carriage. All these samples were previously culture-enriched for pneumococcus and tested for *S. pneumoniae* and pneumococcal serotypes using a qPCR-based protocol (5). On top of that, paired nasopharyngeal samples collected from the same individuals were tested using the WHO-recommended method of cultured pneumococcal isolate serotyping with Quellung (5, 12). When samples of culture-enriched saliva were tested in duplex dPCR targeting *lytA* and *piaB,* the percentage linkage detected was lower than expected based on tests with pneumococcal cell suspensions (Fig. S2A and B). It implied that the number of viable pneumococcal cells plummeted in these samples due to either fratricide or bactericidal effects of co-cultured bacteria in saliva cultures incubated in the original study for a relatively long period of overnight (37). To test for this, we compared different culture incubation times for the *S. pneumoniae* Hungary 19A-6 strain. Linkage percentage was observed to be highest after 6 h (Fig. S2C and D). Accordingly, fresh 6-h CBA cultures were prepared from *n* = 43 study samples of culture-enriched saliva previously identified as positive for *S. pneumoniae* by qPCR (Fig. 3A and B), with C_q_ values ranging from 20 to 36 C_q_ (among original samples), or equivalent of 250 to 0.05 cp/µL. Linkage between *piaB* and *lytA* was observed in 26 of 43 (60.5%) 6-h culture samples (Fig. 3C), with linkage percentages ranging from 13.3% to 83.3% (mean 34.9%; Table S4), and with samples that tested negative for linkage typically displaying quantification of *piaB* and/or *lytA* below the LoD_95_.

### dPCR allows for molecular serotyping of saliva study samples positive for pneumococcus

**Fig 4:**
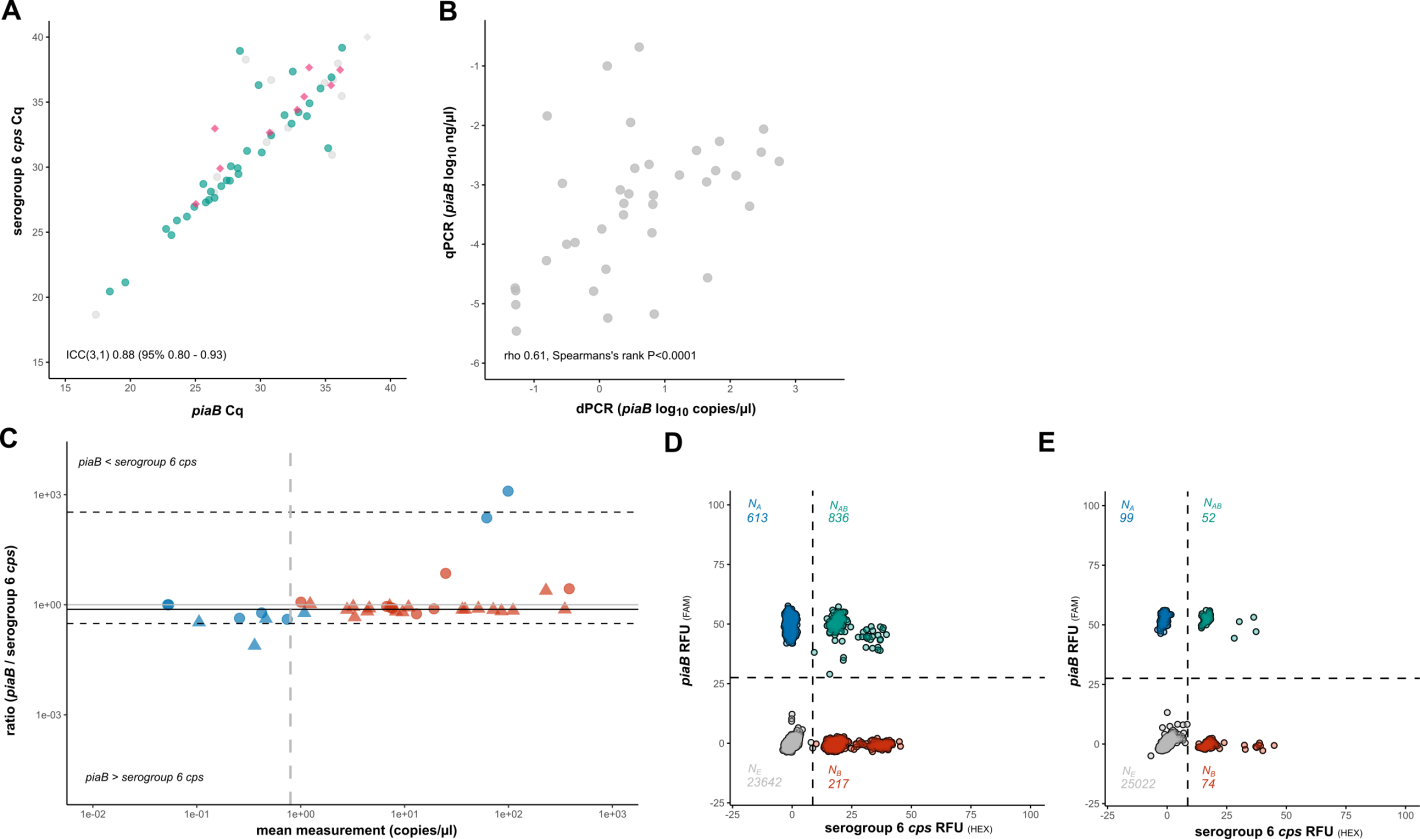
dPCR-based linkage analysis enables accurate molecular detection of serogroup 6 *S. pneumoniae* in culture-enriched saliva samples. (**A**) qPCR-based quantification (in C_q_) of *piaB* and serogroup 6 *cps* in DNA extracts of culture-enriched saliva study samples. Samples from children and adults that were selected for dPCR-based linkage analysis are highlighted in green and pink, respectively. The remaining samples are colored gray. (**B**) The correlation between cultures suggests that pneumococcal presence, as measured by *piaB*, was retained in saliva cultures after preparation of 6-h cultures (dPCR) from the original overnight cultures (qPCR). (**C**) Non-parametric Bland-Altman plot of dPCR-based quantification (in copies/µL) *piaB* and serogroup 6 *cps* in culture-enriched cell suspensions, samples positive for linkage between *piaB* and serogroup 6 *cps* are highlighted in red, and negative samples are highlighted in blue. Circles and triangles indicate samples from culture-negative and culture-positive individuals, respectively. Vertical line indicates the LoD_95_, dashed horizontal lines the limits of agreement, and solid line the bias (median difference between paired *piaB* and serogroup 6 *cps* measurements). (**D**) An example of a 2D dPCR plot wherein linkage is observed between *piaB* and serogroup 6 *cps*. An example of a high-abundance sample. (**E**) An example of a 2D dPCR plot wherein linkage is observed between *piaB* and serogroup 6 *cps*. An example of a low-abundance sample.

Six-hour CBA cultures were prepared from a subset of *n* = 43 culture-enriched saliva study samples that had previously tested positive by qPCR (Fig. 4A) for pneumococcal molecular targets and, in the assay targeting *wciP* gene, as positive for serogroup 6 *cps*. These include *n* = 22 saliva samples from individuals (21 children and one adult) positive for serogroup 6 *S. pneumoniae* by the WHO-recommended conventional culture method applied to nasopharyngeal swab. Linkage between *piaB* and the serogroup 6 *cps* was detected, as long as the quantification of one of the two targets was not below the LoD_95_ (Fig. 4C). In total, 26 of 43 (60.5%) samples were deemed positive for serogroup 6 pneumococci based on the detected linkage estimated by *piaB*. Eighteen (69.2% of 26) linkage-positive saliva samples were from individuals who were also positive for serogroup 6 pneumococci by Quellung from nasopharyngeal cultures. Only a single sample was negative for linkage yet positive for quantification of the LoD_95_ for both targets. This sample exhibited RFU amplitude indicative of serotype 6A (data not shown), suggesting this sample may harbor a *piaB*-deficient *S. pneumoniae* strain, although an oral *Streptococcus* with serogroup 6 *cps* could not be excluded.

### dPCR linkage analysis resolves pneumococcal serotype signature from commensal interference

**Fig 5:**
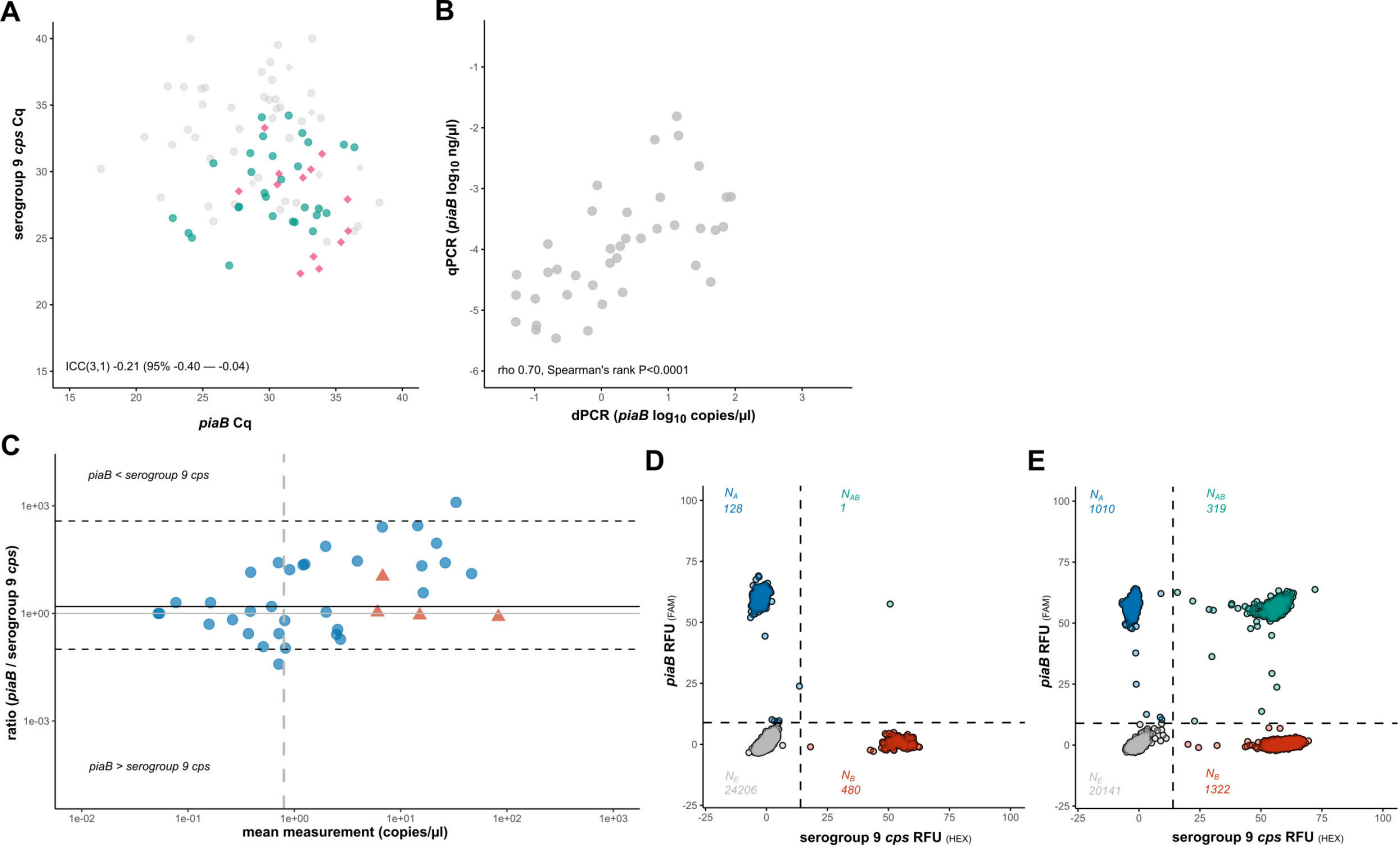
dPCR-based linkage analysis can distinguish between serogroup 9 *S. pneumoniae* and serogroup 9 signal from oral streptococci. (**A**) qPCR-based quantification (in C_q_) of *piaB* and serogroup 9 *cps* in DNA extracts of culture-enriched saliva study samples. Samples from children and adults that were selected for dPCR-based linkage analysis are highlighted in green and pink, respectively. The remaining samples are colored gray. (**B**) The correlation between cultures suggests that pneumococcal presence, as measured by *piaB*, was retained in saliva cultures after preparation of 6-h cultures (dPCR) from the original overnight cultures (qPCR). (**C**) Non-parametric Bland-Altman plot of dPCR-based quantification (in copies/µL) *piaB* and serogroup 9 *cps* in culture-enriched cell suspensions, samples positive for linkage between *piaB* and serogroup 9 *cps* are highlighted in red, and negative samples are highlighted in blue. Circles and triangles indicate samples from culture-negative and culture-positive individuals, respectively. The vertical line indicates the LoD_95_, dashed horizontal lines the limits of agreement, and the solid line the bias (median difference between paired *piaB* and serogroup 9 *cps* measurements). (**D**) An example of a 2D dPCR plot wherein no linkage is observed between *piaB* and serogroup 9 *cps,* yet both targets were quantified. (**E**) An example of a 2D dPCR plot wherein linkage is observed between *piaB* and serogroup 9 *cps*, high-abundance sample.

Next, linkage analysis between *piaB* and serogroup 9 *cps* was assessed for a subset of 43 saliva study samples from *n* = 29 children and *n* = 13 parents that were previously identified as positive for both targets by qPCR (Fig. 5A). The set included samples from four individuals (three children and one adult) whose paired nasopharyngeal samples were culture-positive for serotype 9N *S. pneumoniae*. Linkage was detected in four samples (Fig. 5C), all from the abovementioned individuals from whom serotype 9N pneumococci were cultured and typed by Quellung. Of the remaining 39 samples negative for linkage between *piaB* and serogroup 9 *cps,* seven were positive for both targets above the LoD_95_. Since the serogroup 9 *cps* assay was among the assays classified as unreliable in oral samples, these seven samples likely contained oral streptococci harboring the sequence targeted in serogroup 9 *cps* assay.

### Demonstrated risk of false-positive vaccine serotype identification in molecular testing of URT samples

**Fig 6:**
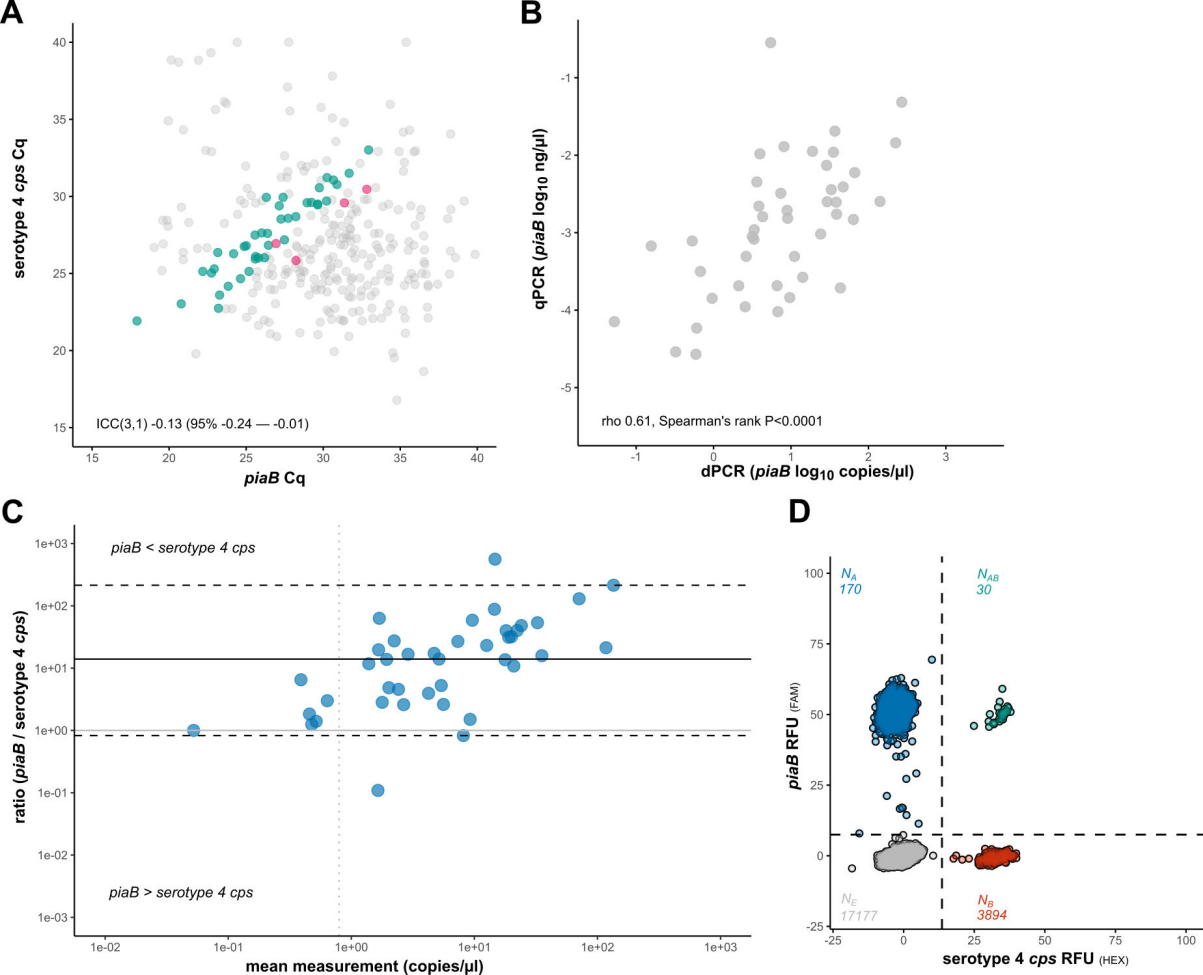
dPCR eliminates false-positive detection of serotype 4, a *cps* that is ubiquitous among oral streptococci. (**A**) qPCR-based quantification (in C_q_) of *piaB* and serotype 4 *cps* in DNA extracts of culture-enriched saliva study samples. Samples from children and adults that were selected for dPCR-based linkage analysis are highlighted in green and pink, respectively. The remaining samples are colored gray. (**B**) The correlation between cultures suggests that pneumococcal presence, as measured by *piaB*, was retained in saliva cultures after preparation of 6-h cultures (dPCR) from the original overnight cultures (qPCR). (**C**) Non-parametric Bland-Altman plot of dPCR-based quantification (in copies/µL) *piaB* and serotype 4 *cps* in culture-enriched cell suspensions, samples positive for linkage between *piaB* and serotype 4 *cps* are highlighted in red, and negative samples are highlighted in blue. The vertical line indicates the LoD_95_, dashed horizontal lines the limits of agreement, and the solid line the bias (median difference between paired *piaB* and serotype 4 *cps* measurements). (**D**) An example of a 2D dPCR plot wherein no linkage is observed between *piaB* and serotype 4 *cps*. In this case, the ratio of double-positive to single-positive partitions was not suggestive of linkage (30 × 17,177 ≈ 170 × 3,894).

To rule out post-PCV circulation of serotype 4 pneumococci (Fig. 6A), we assessed linkage between *piaB* and serotype 4 *cps* in saliva samples from PCV10-vaccinated children and their parents. Eighteen of the forty-three (41.9%) tested samples produced quantification for both *piaB* and serotype 4 *cps* in saliva (Fig. 6C). That was interpreted as widespread circulation of serotype 4 *cps* among oral streptococci. Importantly, none of the samples displayed linkage between the targets, indicating that the signal for serotype 4 *cps* originated from a source other than *S. pneumoniae*.

## DISCUSSION

Despite abundant data on pneumococcal carriage in children, studies in non-paediatric populations remain limited (15). Reliance on nasopharyngeal culture has fostered the misconception that pneumococcal colonization is largely absent in adults, despite a relatively high burden of disease in older adults and early 20th-century studies documenting substantial adult carriage rates using mouse inoculation methods applied to oral samples (38). These and other studies suggest that pneumococcal colonization in adults is primarily confined to oral and oropharyngeal mucosal surfaces (13) and that subgroup variation in diagnostic test performance undermines the sensitivity of nasopharyngeal culture (39). Nonetheless, adult carriage surveys are gaining importance with the rollout of adult vaccination programs.

In highly polymicrobial samples like oral samples, where the relative abundance of the targeted organism is low, culture-based approaches with limited selectivity or electivity often produce discrepancies with culture-independent methods, a longstanding phenomenon known as the “great plate count anomaly” (14). To yield meaningful data, adult surveys must include oral fluids or oropharyngeal swabs combined with molecular methods, as most carriers would otherwise not be identified (40). However, lateral gene transfer (LGT) of capsular genes between *S. pneumoniae* and other members of the *Streptococcus mitis* group, common residents of the oral cavity, can compromise the diagnostic specificity of molecular serotyping in airway samples, as observed in several (though not all) epidemiologically relevant assays (5). Clusters of functionally related genes are especially prone to LGT (41), which may also affect the capsular operon, thereby complicating molecular surveillance. To address this, we evaluated digital PCR for its ability to accurately identify *S. pneumoniae* serotypes in highly polymicrobial URT samples, using linkage analysis between *piaB* and serotype-specific targets.

In this study, we adapted the multiplex single intact cell dPCR (“MuSIC” dPCR) protocol of McMahon et al. for the identification and molecular serotyping of *S. pneumoniae* in upper airway specimens (25). The modified protocol uses a shorter 6-h incubation to maximize the relative abundance of *S. pneumoniae* intact cells in samples. The results presented in this study show that concordant quantification of *piaB* and *lytA* in culture-enriched saliva correlates with the detection of *S. pneumoniae*, thereby providing support for qPCR-based approaches in prior studies (15). Furthermore, linkage analysis enabled differentiation between *S. pneumoniae* serogroup 9 *cps* and non-pneumococcal signatures of the same serogroup within individual samples, allowing for accurate molecular serotyping in settings where serotype/serogroup-specific targets circulate in both *S. pneumoniae* and non-pneumococcal subpopulations.

Consistent with previous studies on pathogenic *Escherichia coli* (25, 26), linkage analysis using DNA extracts proved not feasible for physically distant gene targets. In our case, when using purified *S. pneumoniae* DNA, the spatial separation between targets far exceeded the typical DNA fragment length of DNA extracts, resulting in minimal *piaB/lytA* linkage percentages (−0.8% to 0.3%). To overcome this limitation, we performed linkage analysis on intact cells instead, which yielded substantially higher linkage percentages, ranging from 53% to 71%. Given the low levels of *S. pneumoniae* abundance in oral fluids, the linkage percentage was estimated independently of abundance (5). Linkage percentages below the theoretical maximum of 100% were likely to reflect the quantification of extracellular DNA from permeabilized cells, which, like DNA extracts, did not enable identification of linkage (26). In line with this notion, samples from overnight cultures produced low linkage percentages, ranging from 0.9% to 7.6%, which suggested that quantification in these samples was primarily derived from permeabilized cells. We hypothesized that shorter incubation times might yield higher linkage values, based on unpublished findings indicating that *S. pneumoniae* enrichment with gentamicin on sheep blood agar predominantly occurs within the first 8 h of incubation. Indeed, a 6-h incubation led to a marked increase in linkage in four out of five selected study samples. Further tests with suspensions of pneumococci in mid-log-phase indicated that a 6-h incubation produced maximal linkage compared to longer incubation times. A low percentage of linkage between PCR targets in older cultures was hypothesized to be due to the synthesis of antagonistic compounds during the stationary phase of bacterial growth (37), potentially leading to diminished viability and the accumulation of extracellular (and fragmented) DNA. Accordingly, all study samples were thereafter tested for linkage using a 6-h incubation. Prospective studies aiming to utilize dPCR for linkage analysis should therefore consider the 6-h cultures over overnight cultures.

Given the possibility of PCR-inhibitory compounds in bacterial suspensions leading to “rain” (partitions with intermediate fluorescence) and false-negative results, we evaluated whether extending the number of PCR cycles could improve the identification of linkage between genes. A modest increase in linkage was observed when the thermocycling program was extended to 55 cycles of annealing and extension, compared to 45 cycles. Contrary to previous reports (35), the addition of lysozyme did not further improve linkage detection. Likewise, the addition of bovine serum albumin did not improve linkage detection (data not shown). Thereafter, the analytical sensitivity of the method was evaluated using probit regression analysis of replicate measurements of serially diluted mid-log-phase *S. pneumoniae* suspensions. A LoD_95_ of 1,458 CFU/mL was found, amounting to 0.80 copies/µL.

Accuracy of the method was assessed using mixes of *S. pneumoniae* 9N (*piaB*-positive) with non-pneumococcal serogroup 9 (*piaB-*negative^–^) strains. Linkage between *piaB* and serogroup 9 *cps* was identified, provided that the abundance of the *S. pneumoniae* strain was above the LoD95. The linkage value based on serogroup 9 *cps* reflected the ratio of *S. pneumoniae* 9N and non-pneumococcal serogroup 9 strain, whereas the linkage value based on *piaB* solely reflected the *S. pneumoniae* 9N strain.

A set of 43 culture-enriched saliva samples varying in pneumococcal abundance for both *lytA* and *piaB* were tested with dPCR linkage analysis. Prior analysis of these samples with qPCR had suggested positive conditional dependence between *piaB* and *lytA*, as indicated by near-perfect agreement in paired measurements in Bland-Altman analysis (5). In line with this notion, linkage between *piaB* and *lytA* was observed in culture-enriched saliva, provided that quantification of both targets was above the LoD_95_. Linkage was regarded as evidence of positive conditional dependence between *piaB* and *lytA,* and the co-occurrence of both targets on a bacterial genome was considered specific to encapsulated *S. pneumoniae* (8). This finding provided further support for the utility of the Two-to-Tango approach in guiding the interpretation of carriage surveillance data on polymicrobial samples. Linkage was also identified between *piaB* and serogroup 6 *cps* assays for samples with concordant quantification of both targets, and provided that quantification was above the LoD_95_. In this analysis, the *piaB* assay was used as an *S. pneumoniae*-specific marker, and linkage between both assays was regarded as evidence of a serogroup 6 *cps S. pneumoniae*. The serogroup 6 *cps* assay also produced concordant quantification in prior studies (5), and this, in conjunction with the findings from dPCR-based linkage analysis, suggested that such assays can be reliably interpreted with qPCR.

Next, the serogroup 9 *cps* assay was chosen as qPCR-based surveillance, and nasopharyngeal culture data had suggested simultaneous circulation of *S. pneumoniae* serogroup 9 *cps* and non-pneumococcal *S. mitis* group serogroup 9 *cps*. This assay includes the PCV21 vaccine type (VT) 9N, as well as the PCV7/10/13/15/20 VT 9V. Indeed, dPCR analysis demonstrated that the majority of the saliva samples were negative for linkage between *piaB* and serogroup 9 *cps*, a finding suggestive of a non-pneumococcal origin of sequences detected in the assay targeting serogroup 9 *cps*. Nevertheless, the saliva samples from four individuals, all of which were positive by nasopharyngeal culture for 9N, displayed linkage between targets ranging from 16% to 58%. These observations highlighted the ability of dPCR-based linkage analysis to control diagnostic specificity and differentiate non-pneumococcal serogroup 9 *cps* signatures from pneumococcal serogroup 9 *cps* signatures. As such, the outlined protocol provides a promising methodology for molecular serotyping in studies utilizing culture-independent techniques. This finding bears particular importance to adult carriage studies where oral fluids and oropharyngeal samples are the sample of choice (13, 15). It also demonstrates the risk of false-positive pneumococcal serotyping results when certain precautionary measures are not considered (e.g*.*, assessment of concordance). In our previous studies, qPCR results for such assays were altogether excluded from epidemiologic interpretation (5, 12).

This risk was particularly evident for the serotype 4 *cps* assay. Serotype 4 *S. pneumoniae* is a highly invasive serotype and a PCV7-generation VT that continues to cause pneumococcal disease and is at times linked to localized outbreaks (42, 43). The serotype 4 *cps* PCR assay is in multiple settings associated with widespread circulation of non-pneumococcal *S. mitis* group serotype 4 *cps*. Consequently, molecular serotyping results on oral fluids, as well as nasopharyngeal samples, are often positive for serotype 4 *cps* yet negative for *S. pneumoniae*-specific markers (e.g., *piaB* and *lytA*). This poses a risk for studies using culture-independent molecular serotyping, as several reports of high post-PCV serotype 4 carriage may have been confounded by non-pneumococcal *S. mitis* group serotype 4 *cps* (44–46). By implementing dPCR-based linkage analysis, a simple method of molecular serotyping that does not require DNA extraction, investigators can distinguish *S. pneumoniae* serotype signatures from non-pneumococcal serotype signatures.

**Fig 7:**
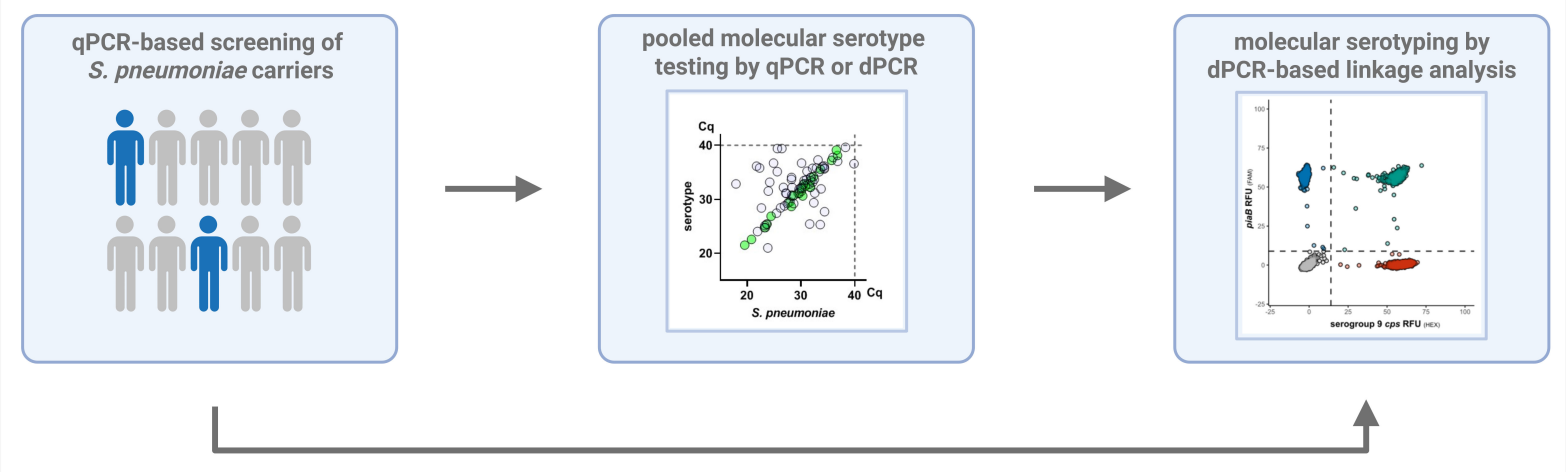
Proposed sample testing workflow. Detection of *S. pneumoniae* in DNA extracts is performed by qPCR, given its lower cost and broad dynamic range. Thereafter, samples classified as *S. pneumoniae* positive are pooled in pools of five samples and tested for a panel of serotype/serogroup-specific assays, either by qPCR or dPCR (e.g*.*, with highly multiplexed dPCR assays). Assays with diagnostic specificity issues can be identified by testing pools of negative samples or via agreement analysis. Such assays (and assays with known specificity issues) are selected for dPCR-based linkage analysis in duplex with *S. pneumoniae* markers (*piaB*/*lytA*) and harvests of cell suspensions (i.e., glycerol-supplemented suspensions). Harvests can be diluted according to qPCR quantification from the corresponding DNA extract to avoid exceeding the dPCR upper detection limit.

Optimized qPCR assays can be easily transferred from qPCR systems to the dPCR platform. On the Qiagen dPCR platform, 26k-well nanoplates can accommodate suspensions of intact bacterial cells, and this platform requires no additional steps, such as droplet generation. In comparison to qPCR, the dynamic range of dPCR is somewhat more limited, with highly concentrated samples requiring prior dilution. To this end, we recommend testing DNA extracts of samples for *S. pneumoniae* with *piaB* and *lytA* by qPCR. Following the identification of *S. pneumoniae* positive samples, these positive samples can be pooled and tested for serotype/serogroup-specific qPCR assays. Individual samples from pools that exhibit serotype/serogroup-specific signal for assays can then be tested individually on the dPCR platform for molecular serotyping (Fig. 7). Although the linkage analysis protocol can technically be applied to any sample, it is only appropriate for samples that have already been confirmed as *S. pneumoniae* positive. At present, we recommend qPCR for routine testing of DNA extracts; however, direct testing by dPCR is also possible. Beyond its application in linkage analysis, dPCR offers additional advantages for molecular surveillance, as ongoing advances in dPCR technology and increased assay multiplexing capacity may improve throughput beyond what is currently possible with qPCR (47).

### Limitations of the study

This study is subject to certain limitations. The protocol requires samples supplemented with glycerol and storage at proper conditions (i.e., −70°C). Only a limited number of assays were tested with the dPCR-based linkage analysis. In presumably rare cases where a sample harbors multiple *S. pneumoniae* serotypes, including the serotype of interest, alongside an oral *Streptococcus* carrying a homologous serotype signature, false-negative results may arise, especially when the target *S. pneumoniae* serotype is present at low abundance. Such samples are expected to show low linkage percentages between the serotype target and *piaB/lytA*, potentially complicating interpretation.

### Conclusion

In conclusion, dPCR-based linkage analysis represents a promising approach for accurate molecular serotyping in polymicrobial upper airway samples. This study demonstrated that dPCR, when applied to intact cells, can reliably detect *S. pneumoniae* in saliva samples from children and adults and can resolve a *S. pneumoniae* serotype signature from those of non-pneumococcal origin. This is especially important for carriage analyses of adults, since pneumococcal colonization in adults is often confined to polymicrobial sites, such as the oral and oropharyngeal mucosa. dPCR has the potential to substantially improve adult carriage surveillance by enabling more accurate molecular detection and serotyping, thereby improving robust evaluation of PCV impact on carriage.

## Data Availability

All data reported in this paper will be shared by the corresponding author upon request. This paper does not report original code. Any additional information required to reanalyze the data reported in this paper is available from the corresponding author upon request. This study did not generate new, unique reagents.

## SUPPLEMENTAL MATERIAL

Figure S1 spectrum.03131-25-s0001.TIFF Probit analysis of 95% limit of detection for linkage assessment.

Figure S2 spectrum.03131-25-s0002.tiff Target quantification and percent linkage in relation to culture incubation times.

Supplemental legends spectrum.03131-25-s0003.docx Descriptive legends for Fig. S1 and S2.

Table S1 spectrum.03131-25-s0004.docx Strain characteristics.

Table S2 spectrum.03131-25-s0005.docx Materials.

Table S3 spectrum.03131-25-s0006.docx Digital PCR primer and probes.

Table S4 spectrum.03131-25-s0007.xlsx Laboratory data of study sample testing.

Table S5 spectrum.03131-25-s0008.xlsx dMIQE details.

ASM does not own the copyrights to Supplemental Material that may be linked to, or accessed through, an article. The authors have granted ASM a non-exclusive, world-wide license to publish the Supplemental Material files. Please contact the corresponding author directly for reuse.
