# Supplementary figures and images for: Digital PCR linkage analysis resolves Streptococcus pneumoniae signature from commensal interference in saliva samples: identifying wolves among sheep in wolf’s clothing
Source: Microbiol Spectr. 2026 Mar 25;14(5):e03131-25. doi: 10.1128/spectrum.03131-25 (PMC13142035; doi:10.1128/spectrum.03131-25)

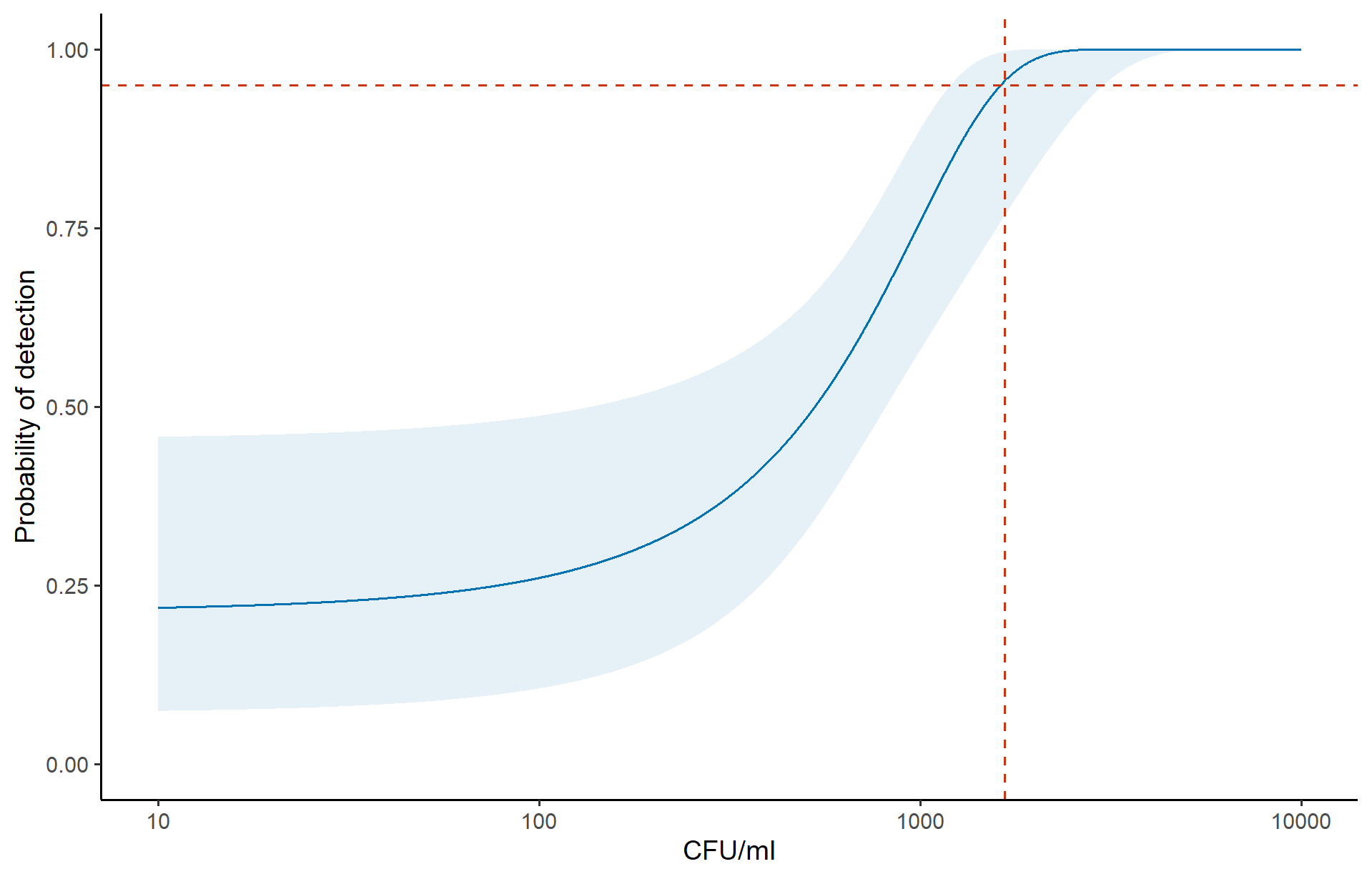

Supplement: Figure S1 — Probit analysis of 95% limit of detection for linkage assessment. [file spectrum.03131-25-s0001.tiff]

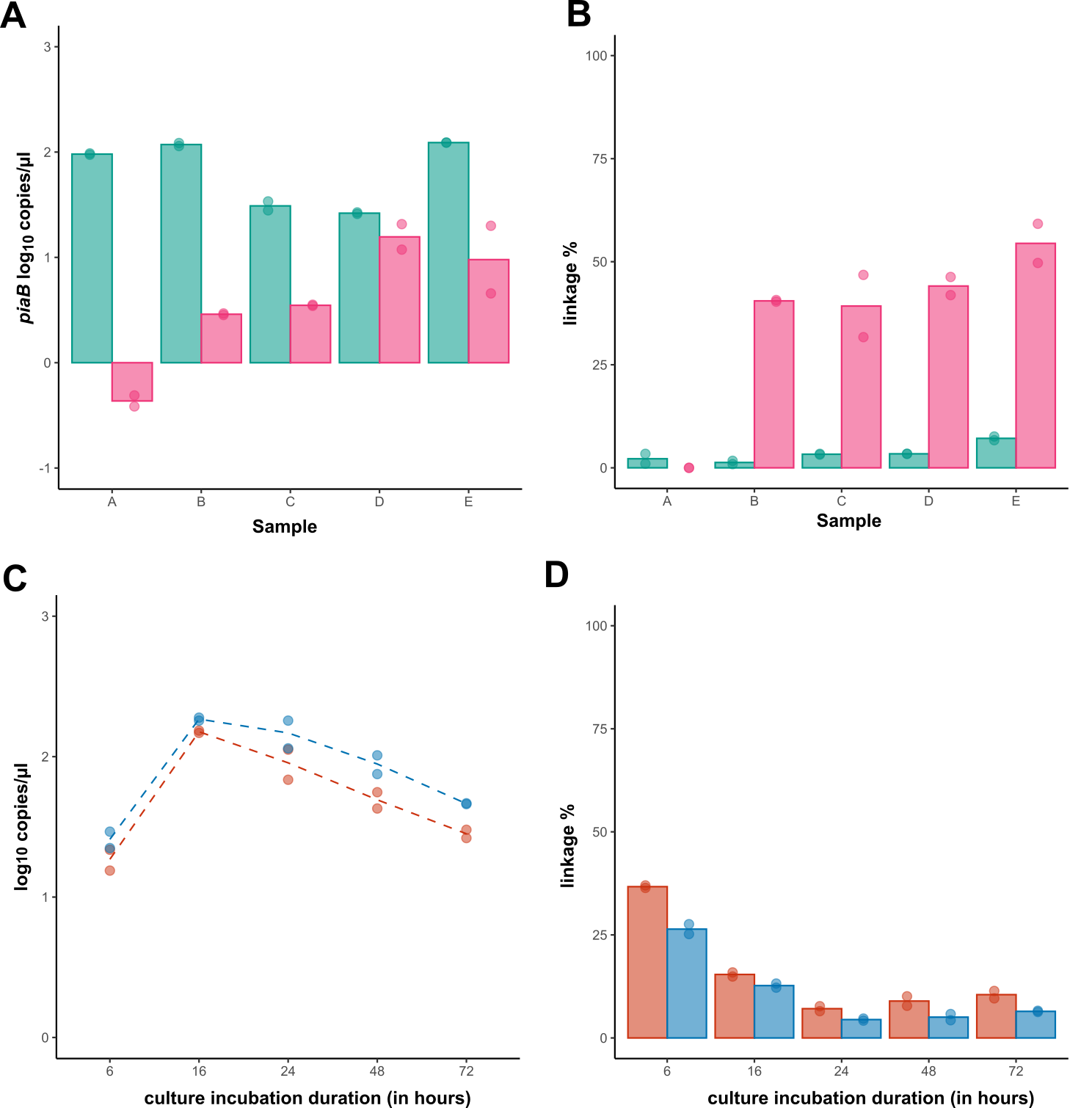

Supplement: Figure S2 — Target quantification and percent linkage in relation to culture incubation times. [file spectrum.03131-25-s0002.tiff]
