# Supplementary material for: Digital PCR linkage analysis resolves Streptococcus pneumoniae signature from commensal interference in saliva samples: identifying wolves among sheep in wolf’s clothing
Source: Microbiol Spectr. 2026 Mar 25;14(5):e03131-25. doi: 10.1128/spectrum.03131-25 (PMC13142035; doi:10.1128/spectrum.03131-25)
Supplement: Supplemental legends — Descriptive legends for Fig. S1 and S2. [file spectrum.03131-25-s0003.docx]

**Figure S1**: **Probit regression analysis of 95% limit of detection (LoD_95_) for the probability of linkage identification between *piaB* and *lytA* given *S. pneumoniae* concentrations (in CFU/ml) in bacterial cell suspensions**. The LoD_95_ corresponded to 1664 CFU/ml, which amounts to 9.2 CFU/reaction or 4.4 cp/reaction.

**Figure S2**: **Target quantification and percentage linkage in relation to culture incubation times**. **A:** log10-transformed concentrations of *piaB,* with 24-hour (green) cultures and 6-hr (pink) cultures. **B:** Percentage linkage of *piaB* among 24-hour (green) and 6-hour (pink) cultures. **C**: log10-transformed concentrations of *piaB* (red) and *lytA* (blue) at different culture incubation durations (in hours). **D**: Percentage linkage of *piaB* (red) and *lyta* (blue) at different culture incubation durations. Among the observed timepoints, linkage percentage of *piaB* and *lytA* was maximal at 6 hours. Accordingly, 6 hours incubation periods were used for linkage analysis on study samples.
