# Supplementary material for: Digital PCR linkage analysis resolves Streptococcus pneumoniae signature from commensal interference in saliva samples: identifying wolves among sheep in wolf’s clothing
Source: Microbiol Spectr. 2026 Mar 25;14(5):e03131-25. doi: 10.1128/spectrum.03131-25 (PMC13142035; doi:10.1128/spectrum.03131-25)
Supplement: Table S1 — Strain characteristics. [file spectrum.03131-25-s0004.docx]

**Table S1: Strain characteristics**

| **Strain name** | **Catalase** | **Bile solubility test** | **Optochin sensitivity** | ***lytA^a^*** | ***piaB^a^*** | **Quellung** | ***cps^a^*** |
| --- | --- | --- | --- | --- | --- | --- | --- |
| Hungary 19A-6 | negative | soluble | sensitive | positive | positive | 19A | serotype 19A |
| P2007-1850 | negative | soluble | sensitive | positive | positive | 9A | serogroup 9 |
| Sm226001019702 | negative | insoluble | resistant | negative | negative | ND | serogroup 9 |
| PI2018-0103 | negative | soluble | sensitive | positive | positive- | 6A | serogroup 6 |
| PI2013-2681 | negative | soluble | sensitive | positive | positive | 6C | serogroup 6 |
| PI2018-0984 | negative | soluble | sensitive | positive | positive | 4 | serotype 4 |

a: Tested by qPCR; ND: not determined.
