# Supplementary material for: Digital PCR linkage analysis resolves Streptococcus pneumoniae signature from commensal interference in saliva samples: identifying wolves among sheep in wolf’s clothing
Source: Microbiol Spectr. 2026 Mar 25;14(5):e03131-25. doi: 10.1128/spectrum.03131-25 (PMC13142035; doi:10.1128/spectrum.03131-25)
Supplement: Table S2 — Materials. [file spectrum.03131-25-s0005.docx]

| REAGENT or RESOURCE | SOURCE | IDENTIFIER |
| --- | --- | --- |
| Bacterial and virus strains | | |
| *Streptococcus pneumoniae* Hungary 19A-6 (serotype 19A) | McGee *et al*. [44] | HUN663 (ATCC 700673) |
| *Streptococcus pneumoniae* (serotype 9A) | This study | P2007-1850 |
| *Streptococcus* indeterminatus (harbors *cps* with sequence homology to *S. pneumoniae* serogroup 9 *cps*) | This study | Sm226001019702 |
| *Streptococcus pneumoniae* (serotype 6A) | This study | PI2018-0103 |
| *Streptococcus pneumoniae* (serotype 6C) | This study | PI2013-2681 |
| *Streptococcus pneumoniae* (serotype 4) | This study | PI2018-0984 |
| Biological samples |  |  |
| Saliva samples | Vissers *et al.* [20] | N/A |
| Chemicals, peptides, and recombinant proteins | | |
| QIAcuity Probe mastermix | Qiagen | #250101 |
| SensiFAST Probe No-ROX Kit | Bioline | #BIO-86050 |
| Brain Heart Infusion broth (dehydrated) | Oxoid | #CM1135 |
| yeast extract | Oxoid | #LP0021B |
| Aqua B. Braun | B. Braun | #0082479E |
| Oligonucleotides | | |
| *piaB* Fw (5’-CATTGGTGGCTTAGTAAGTGCAA-3’) | Eurogentec | N/A |
| *piaB* Rv (5’-TACTAACACAAGTTCCTGATAAGGCAAGT-3’) | Eurogentec | N/A |
| *piaB* P (5’-6FAM-TGTAAGCGGAAAAGCAGGCCTTACCC-BHQ1-3’) | Eurogentec | N/A |
| *lytA* Fw (5’-ACGCAATCTAGCAGATGAAGCA-3’) | Eurogentec | N/A |
| *lytA* Rv (5’-TCGTGCGTTTTAATTCCAGCT-3’) | Eurogentec | N/A |
| *lytA* P (HEX-GCCGAAAACGCTTGATACAGGGAG-BHQ1-3’) | Eurogentec | N/A |
| 6A/B/C/D (serogroup 6 *cps*) Fw (5’-AAGTTTGCACTAGAGTATGGGAAGGT-3’) | Eurogentec | N/A |
| 6A/B/C/D (serogroup 6 *cps*) Rv (5’-ACATTATGTCCRTGTCTTCGATACAAG-3’) | Eurogentec | N/A |
| 6A/B/C/D (serogroup 6 *cps*) P (5’-HEX-TGTTCTGCCCTGAGCAACTGG-BHQ1-3’) | Eurogentec | N/A |
| 9A/L/N/V (serogroup 9 *cps*) Fw (5’-TGGAATGGGCAAAGGGTAGTA-3’) | Eurogentec | N/A |
| 9A/L/N/V (serogroup 9 *cps*) Rv (5’-TCGGTTCCCCAAGATTTTCTC-3’) | Eurogentec | N/A |
| 9A/L/N/V (serogroup 9 *cps*) P (5’-HEX-TTAATCATGCTAACGGCTCATCGA-BHQ1-3’) | Eurogentec | N/A |
| 4 (serotype 4 *cps*) Fw (5’-TGGGATGACATTTCTACGCACTA-3’) | Eurogentec | N/A |
| 4 (serotype 4 *cps*) Rv (5’-CCGTCGCTGATGCTTTATCA-3’) | Eurogentec | N/A |
| 4 (serotype 4 *cps*) P (5’-HEX-TCCTATTGGATGGTTAGTTGGTGA- BHQ1-3’) | Eurogentec | N/A |
| Other | | |
| DNeasy Blood & Tissue kit | Qiagen | #69506 |
| QIAcuity One 2-plex dPCR instrument | Qiagen | #911000 |
| QIAcuity 26K 24-well nanoplate | Qiagen | #250001 |
| PCR plate (for mixing samples with reaction mix) | Greiner | #652290 |
| QIAcuity Nanoplate seal | Qiagen | #250099 |
| LightCycler480 qPCR instrument II | Roche | #05015278001 |
| Digital Minimum Information for Publication of Quantitative Real-Time PCR Experiments (dMIQE) guidelines, **see Table S4** | dMIQE Group & Huggett (2020) | Digital MIQE guidelines |

List of laboratory reagents and materials used in the study.
