# Supplementary material for: Digital PCR linkage analysis resolves Streptococcus pneumoniae signature from commensal interference in saliva samples: identifying wolves among sheep in wolf’s clothing
Source: Microbiol Spectr. 2026 Mar 25;14(5):e03131-25. doi: 10.1128/spectrum.03131-25 (PMC13142035; doi:10.1128/spectrum.03131-25)
Supplement: Table S3 — Digital PCR primer and probes. [file spectrum.03131-25-s0006.docx]

**Table S2**: digital PCR primer and probes

| **Assay** | **Gene** | **Sequence (5’-3’)** | **Amplicon size** | **Oligonucleotide**  **Concentration (nM)** | **Imaging**  **settings** | **RFU**  **threshold^†^** | **Duplex** | **Assay design**  **reference** |
| --- | --- | --- | --- | --- | --- | --- | --- | --- |
| *piaB-*Fw | *piaB* | 5’-CATTGGTGGCTTAGTAAGTGCAA-3’ | 104 bp | 200 | exposure 500  gain 6 | 11.98  27.55  8.93  7.47 | *piaB-lytA*  *piaB-6ABCD*  *piaB-9ALNV*  *piaB-4* | Trzcinski  *et al.* 2013 [22] |
| *piaB-*Rv |  | 5’-TACTAACACAAGTTCCTGATAAGGCAAGT-3’ |  | 175 |  |  |  |  |
| *piaB*-P |  | 5’-6FAM-TGTAAGCGGAAAAGCAGGCCTTACCC-BHQ1-3’ |  | 200 |  |  |  |  |
| *lytA*-Fw | *lytA* | 5’-ACGCAATCTAGCAGATGAAGCA-3’ | 75 bp | 200 | exposure 750  gain 9 |  | *piaB-lytA* | Carvalho  *et al.* 2007 [23] |
| *lytA*-Rv |  | 5’-TCGTGCGTTTTAATTCCAGCT-3’ |  | 225 |  | 14.27 |  |  |
| *lytA*-P |  | HEX-GCCGAAAACGCTTGATACAGGGAG-BHQ1-3’ |  | 100 |  |  |  |  |
| 6ABCD-Fw | *wciP* | 5’-AAGTTTGCACTAGAGTATGGGAAGGT-3’ | 77 bp | 200 | exposure 750  gain 9 |  | *piaB-6ABCD* | Azzari  *et al.* 2010 [45] |
| 6ABCD-Rv |  | 5’-ACATTATGTCCRTGTCTTCGATACAAG-3’ |  | 200 |  | 8.63 |  |  |
| 6ABCD-P |  | 5’-HEX-TGTTCTGCCCTGAGCAACTGG-BHQ1-3’ |  | 125 |  |  |  |  |
| 9ALNV-Fw | *mnaA* | 5’-TGGAATGGGCAAAGGGTAGTA-3’ | 69 bp | 200 | exposure 500  gain 9 |  | *piaB-9ALNV* | Azzari  *et al.* 2010 [45] |
| 9ALNV-Rv |  | 5’-TCGGTTCCCCAAGATTTTCTC-3’ |  | 250 |  | 8.93 |  |  |
| 9ALNV-P |  | 5’-HEX-TTAATCATGCTAACGGCTCATCGA-BHQ1-3’ |  | 175 |  |  |  |  |
| 4-Fw | *wzy* | 5’-TGGGATGACATTTCTACGCACTA-3’ | 86 bp | 200 | exposure 500  gain 6 |  | *piaB-4* | Azzari  *et al.* 2010 [45] |
| 4-Rv |  | 5’-CCGTCGCTGATGCTTTATCA-3’ |  | 200 |  | 13.58 |  |  |
| 4-P |  | 5’-HEX-TCCTATTGGATGGTTAGTTGGTGA- BHQ1-3’ |  | 200 |  |  |  |  |

†: Following baseline correction.
